# Maintenance and Development of Social Connection by People with Long-term Conditions: A Qualitative Study

**DOI:** 10.3390/ijerph16111875

**Published:** 2019-05-28

**Authors:** Amanda Wilkinson, Lucy Bowen, Elias Gustavsson, Simon Håkansson, Nicole Littleton, James McCormick, Michelle Thompson, Hilda Mulligan

**Affiliations:** 1 Centre for Health, Activity, and Rehabilitation Research, School of Physiotherapy, University of Otago, P.O. Box 56, Dunedin 9054, New Zealand; mandy.wilkinson@otago.ac.nz (A.W.); lucybowen97@gmail.com (L.B.); n.littleton@windowslive.com (N.L.); jgmccormick94@gmail.com (J.M.); thompsonmichelle96@gmail.com (M.T.); 2 Department of Community Medicine and Rehabilitation, Umeå University, 901 87 Umeå, Sweden; eliasgustavsson@hotmail.com (E.G.); hakansson.simon@outlook.com (S.H.)

**Keywords:** social connection, long-term conditions, health, qualitative research

## Abstract

Social connection is important for people’s health and well-being. Social isolation arising from a lack of meaningful connection with others can result in deterioration of well-being with negative consequences for health. For people living with multiple long-term conditions, the building and maintaining of social connection may be challenging. The aim of this study was to explore with people with long-term conditions how they perceive they maintain and develop social connections. We undertook semi-structured interviews with seventeen adults, and analyzed the data for themes. Themes were “Meaningful connection”, “Wherewithal for social connection” and “Impact of a major change in life course”. The findings suggest that social connection is valued, and facilitates meaningful ways to reciprocate support with others, thus enabling access to knowledge and resources for better health and well-being. However, people with long-term conditions can experience challenges to developing and maintaining social connectedness after a major change in life course. We suggest that healthcare providers are well placed to facilitate ways for people with long-term conditions to socially connect with others in their neighbourhood and community, and that this in particular be attended to after a major life change.

## 1. Introduction

Current research indicates that social connectedness correlates with people’s health status [1,2]. Indeed, social connectedness has been found to be associated with both mental and physical health benefits [1,3,4]. Feeling part of social activity has been shown to reduce stress, and enhance self-esteem and cognition, thereby improving people’s health and well-being [2]. However, social isolation, because of a lack of social connection, can result in a deterioration in well-being with negative consequence on health [5]. Very recently, some governmental bodies are now beginning to acknowledge the role that connections with friends, family and community members can have in helping people feel that they belong and have a role to play in society [6,7,8]. 

The concept of social connection has many definitions [9]. The term “social connection” refers to a person’s positively experienced social relationships [1] as an important aspect of their life [10]. Social connection encompasses the relationships, networks or links that people have with other people or groups, whether these be with neighbours, family, friends, sporting or social groups. “Social connection” is about the “people we know; the friends we confide in, the family we belong to and the community we live in” ([11], p. 219). Attachment is the term used to describe the first “social” connection between an infant and its mother. How the relationship develops will affect the infant’s future interactions both with the mother and with others [12]. A wider and more general term for social connection, often used in the clinical literature, is social support. Social support encompasses not only practical aid and informational support, but could also include the building of self-esteem through a feeling of belonging [13]. Another term referring to social connection is “belongingness”, which describes a basic human need to feel accepted and to feel part of something bigger, and can/tends to include intimate relationships [1]. Finally, the term social connection can refer to any and all connections made with others. Therefore, social connection could encompass everything from connections with families and friends, to connections made at work or through different activities. The ability to develop social connections is also dependent on the context of a situation and an individual’s experience of social interactions with others in that situation. These elements of connectedness fit with the concept of connectedness described by Phillips-Salimi and colleagues through an analysis of a wide variety of literature from disciplines including education, medicine, nursing, psychology and public health examining connectedness across the lifespan [14]. For this study we have defined the idea of social connection as an individual’s perception of meaningful and reciprocal connection or emotional interactions in relationship with others [9]. Such relationships are developed via participation in family, social, and community activities that are perceived as meaningful to an individual and that can be within one’s neighbourhood or other context for that individual. 

For people who live with multiple long-term conditions, the ability to maintain existing social connections and networks, and to develop new supportive networks may be difficult [15,16,17]. Reasons for this are many and can relate to the effect of symptoms (physical, emotional, mental) arising from long-term condition/s on a person’s body [18] and self [19], the out-of-pocket cost of living with long-term conditions [20], characteristics of the built environment including its walkability [21], or indeed the underlying attitude of healthcare providers about long-term condition management [22]. These aspects or a combination of these can have a detrimental effect on an individual’s overall well-being, health and physical activity levels [5,23,24]. 

Most of the literature around maintenance and development of social connection by people with long-term conditions is of a quantitative nature with limited exploration of this concept from the personal perspective. The aim of this study was therefore to explore with people with long-term conditions how they maintain and develop social connections. The intent was to be able to inform how healthcare professionals could help to improve the health and well-being of people with long-term conditions by increasing social connectedness within this group of the population.

## 2. Materials and Methods

This study used a qualitative descriptive approach [25,26]. We chose this approach to facilitate exploration of the perspectives of people living with long-term conditions about how they perceive they maintain and develop social connection with others [27]. This study contributed to a larger research project titled WellConnectedNZ^™^. The WellConnectedNZ^™^ project explored ways to facilitate people with long-term conditions to access community support through improving social connectedness, physical activity, and health literacy in order to better manage their health and well-being. The WellConnectedNZ^™^ research project was approved by NZ Health and Disability Committee (17/NTB/2016). We have used the Consolidated Criteria for Reporting Qualitative Research (COREQ) [28] as our guideline for reporting of this study and to show transparency of the study for the reader. 

We recruited participants (aged 18 and over) with long-term conditions through electronic and newsletter flyers sent via organizations that support people with long-term conditions (for example the local multiple sclerosis, Parkinson’s, and respiratory conditions organizations), direct invitation to attendees at coffee and/or exercise groups for people with long-term conditions, and via word of mouth (snowball sampling by participants). Interested individuals contacted one of the researchers (AW), were provided with study information and any questions about the research were answered. Participants were recruited from the same geographical area within a large metropolitan city in New Zealand that the WellConnectedNZ^™^ team chose for their larger project. This area was chosen specifically due to its lower socio-economic status compared to other parts of the metropolitan city. Additionally, people in the study area were severely affected by a natural disaster of a series of earthquakes, and at the time of the study, did not inhabit a built environment that matched pre-earthquake conditions. All participants provided signed consent to be interviewed. 

Pairs of researchers (L.B., N.L., J.McC., M.T., E.G., S.H. under the supervision of A.W. & H.M. who are experienced researchers) conducted semi-structured individual interviews (Table 1) at a suitable time and place for participants. This was mostly their homes. Interviews lasted between 20–90 minutes. Two participants had a spouse or partner present during the interview. Prior to the interview commencing, verbal consent to being digitally recorded was obtained from each participant. We also collected demographic data consisting of age, gender, ethnicity and self-reported long-term condition diagnoses. 

Members of the team transcribed the interviews verbatim and removed all personally identifying information. Transcriptions were then double checked against the original recording by another team member. We analyzed interview data for themes using an inductive approach [29]. Each team member independently read and coded half of the transcripts. Team members then came together and reached consensus on each code and developed a coding template which listed and defined each code. This template was then used to independently code the remaining transcripts. Any new codes, were debated as a group before being added to the coding template. Codes and representative quotes from the transcripts were tabulated. Many group discussions were held to discuss grouping the codes into categories, subthemes and themes, and to define the subthemes and themes. Data saturation was achieved by interview 10, however all transcripts were analyzed. 

## 3. Results

Seventeen people (F = 12; M = 5) with an age range of 50–79 years participated in this study (Table 2). Participants reported being diagnosed with one to four long-term conditions each, with most participants reporting more than two conditions. Nine participants were retired and five were unemployed. Five participants lived alone. 

Analysis of the interview transcripts resulted in three major themes of “Meaningful connection”, “Wherewithal for social connection” and “Impact of a major change in life course”. These are described below with quotes from participant interviews used to illustrate the themes and subthemes (Table 3).

### 3.1. Theme 1: Meaningful Connection

The theme of “Meaningful connection” identifies why and with whom participants developed meaningful relationships with others. Participants connected with other people for a reason, such as a common interest, *I ride motorbikes, I’m in a club (Participant 14),* or to help other people as a way of “giving back” because of help they had personally received, and for which they were grateful. *I’m going to request that I can continue to go to them [the exercise class] but to volunteer to make teas and coffees or whatever, doing things like this [volunteering], [it’s] just sort of giving back really. It was given to me freely and I wanna give it [back] freely (Participant 4).* Other participants sought connection because they themselves needed support and assistance. *I actually went in there [a community church] one day cos I was very, extremely desperate [because I had lost my job] and yeah they’ve helped me immensely. They [helped me] with budgeting advice (Participant 1)* or because they knew that getting out and doing something with others was beneficial for themselves. *I loved it [exercise with a group]. I had a really good time and felt safe and happy. … Yeah I was a bit worried at first, cos I really don’t like group situations. … I’m looking forward to going back to the next [class] (Participant 17).*

Participants derived connections from people close to them, or from other people in their neighbourhood. Often it seemed that family provided the reason for getting out and connecting with others *[Our] son sings [at the club] so we go out there and if we’re fit enough to get up and have a dance, we will (Participant 16 and wife).* For others, their family seemed to be the source of social connection *my kids [visit], yeah, they keep an eye on me especially [after death of his spouse] (Participant 9).* Friends provided the impetus for social connection by picking participants up for outings. *My friend takes me, she lives down the road. I met her through [something else we attended together] and she takes me shopping every week (Participant 17).* Neighbours were often mentioned as a source of social connection for participants *[my] neighbours, having cups of tea together, bringing in their washing when it rains, ringing them all the time [and talking], saying ‘gidday’ [hello] when you’re in the garden (Participant 13).* One participant spoke of the connection he derived from talking with people in his neighbourhood such as staff at the grocery store, or at his medical center. *Down at the supermarket, there’s some good check-out girls [staff] down there, you can have a good talk with them. … When I go to the doctor, I have a yarn with them [nurses] and that is a good connection. He [the doctor] also likes sitting there talking with me (Participant 12).*

### 3.2. Theme 2: Wherewithal for Social Connection

The second theme of “Wherewithal for social connection” captures some of the intrinsic person factors and extrinsic factors that appeared to impact on participants’ ability to maintain or develop social connections. Intrinsic person factors included participants’ mind-set about connecting with others that either facilitated social connection or did not. One participant seemed to have a mind-set that meant she preferred to *get out and talk to people (Participant 13)* and she actively sought ways to meet new people. *When we [moved] there, I knocked on all the doors and introduced myself so they all knew who I was (Participant 13).* Others described how they no longer socialized a lot and put this down to ageing. *[When I was younger] I was doing things with [others] … I don’t really socialize a lot [now] but most people our age don’t (Participant 2).*

Participants identified that the symptoms they experienced in relation to their long-term condition had curtailed their ability to get out and about, *I don’t get out much because of my illness. I get so short of breath. I try to go to [visit] my parents when I can, they’re not far from here. [It is] too far to walk, but I try and bus [there] when I can (Participant 17),* work fulltime *I’d love to get back to [fulltime] work again. … But I can only do two or three hours a day now [because of the dusty environment at workplaces over 40 years of working]. You can’t get a [fulltime] job and only do two to three hours [a day] (Participant 12)*, or participate in their hobbies and thus maintain social connection with others. *I stopped playing golf because you have to be able to move around the course quite quickly. I can’t walk very fast now, and this [holds up other players] (Participant 1).*

A further potential intrinsic person factor to maintaining or developing connection with others was participants’ reluctance to use or their dislike of the use of technology to connect with others. Many of the participants preferred face to face exchanges *I don’t pay bills [via] the internet, cos I want to get out and talk to people (Participant 13)* or preferred to talk with people on the phone instead of using internet mediated platforms. *My son’s in Australia so he set me up with a Facebook page. I don’t know how to access it. Phone calls are better (Participant 12).* Others did not own a cell phone and did not use a computer because they were of the *old school [and] scared that [they] were not competent enough [to use a computer] (Participant 16).* However, there were participants who felt comfortable enough using technology (cell phone, tablet, or computer) and therefore used technology to connect with meaningful others. *My kids would tell you that I am not [in tune with technology] but compared to some people of my age, I am. One of my sons [gave] me a new phone for Christmas and it drives me crazy. I prefer my funny old one. However I am getting [used to the new one]. I prefer the grandchildren to teach me rather than the kids. Grandchildren don’t mind [showing me] a thousand times over (laughs) (Participant 10).*

Extrinsic factors that impacted on participants’ ability to connect with others were finances, the weather, and proximity to amenities such as the supermarket. Three participants reported that a lack of discretionary money stopped them from participating in activities that allowed for social connection with others. One participant no longer socialized at the pub *because they [drinks and food] were too expensive (Participant 12),* and another, had stopped playing golf because she could no longer afford the green fees and club membership after giving up work because of her long-term condition. *It [golf] is very expensive, and obviously I can’t afford that now (Participant 1).* A further participant had the opportunity to connect with others at a local library, but because of an outstanding fine with the library that she could not afford to pay, no longer went to the library. *I used to go to the library, but I’ve got a fine. I’ve gotta go down and sort it out, but I can’t afford it (Participant 17).*

The weather had a particular impact on participants with breathing difficulties, for example, temperature changes. Not every day is the same. Where I can get out and walk for half an hour one day, I couldn’t another because of the temperature change. [My breathing] is affected by the cold (Participant 4). For others, the wind impacted on their feeling of being able to breathe. The wind, it gets in my face and I can’t breathe and I get short of breath. Like, today is lovely, so I could go for a walk, but yesterday we went to the grave [of a friend] and I couldn’t stay [and be with others] because it was too windy. I had to leave (Participant 17).

Participants identified the effect of the logistics of travelling to amenities such as the supermarket, hospital or groups they attended. Some of the participants lived where they were able to walk or use public transport to get out and about for particular activities, but for other activities, they relied on others for transport. *On a good day I can walk to the [nearest] supermarket. But if it’s a bad day [health wise], I get the bus to the next supermarket. That way I only have to walk a small way there and back. … I used to rely on the buses to go to my Tuesday group, but somebody picks me up on a Tuesday now … it was too far to walk from the bus stop to the community center [where the group meets] (Participant 15).*

As an illustration, for those participants who had sufficient wherewithal for social connection the outcome was a full and connected daily or weekly schedule. Participant 10 epitomized this and her enjoyment of being so involved. *Gosh, do you really want to know blow by blow? (Laughter) Monday morning is … Tuesday morning I help with … Tuesday afternoon I go to …. Wednesday morning is … I also run a lunch first Wednesday of every month … Thursdays I run another group … Friday I go to … I also sit on committees and working groups. So yes, extremely busy, but I love it (Participant 10).* In contrast, other participants had very little in their weekly schedule. In fact, Participant 3 described her usual week as *“boring”* because the only thing she reportedly “did” outside of the home during the week that facilitated connection with other people was attend a scheduled rehabilitative exercise class. 

### 3.3. Theme 3: Impact of a Major Change in Life Course

The third and final theme “Impact of a major change in life course”, had three contributing sub-themes. These were “Reasons for major change”, “Reflections” and “Resignation, resilience and coping”. The first sub-theme encapsulates reasons for the major changes participants had experienced in their lives that impacted on their ability to socially connect with others. One participant who had retired, suggested it was much easier to connect with others when he was working. *When I was working, that was completely different. You always had someone to talk to, your workmates to get through the day with, and you met new people all the time, you know. Since I haven’t been working, it’s been a lot harder to meet people (Participant 12).* For others, the diagnosis of a long-term condition had impacted on their emotional or physical capacity for social connection as seen in these quotes. *I had a terrible time coming to terms with that [diagnosis of chronic obstructive pulmonary disease] because I know the disease is irreversible (Participant 4). I did have a life before everything turned to crap [got unwell]. I used to be able to [go out to] socialise [before my health declined and I was diagnosed with long-term conditions] (Participant 6).* Participants also talked about the impact the death of a spouse had on their life because of the feeling of connectedness with another human being that the spouse had provided. *When [husband] was here, we used to just sit [together], he’d watch telly [television] and I’d knit. We’d go out and do the garden together. It is too much for me now and I don’t like being out there on my own. I’d rather have someone to talk to. Loneliness. Loneliness is the worst part of it. It’s not being alone, being alone doesn’t worry me. It’s just being lonely (Participant 3).* Many of the participants talked about how their neighbourhood changed after a natural disaster (a sequence of earthquakes) and the impact this had on their feeling of connection with others in their area. *I’ve lived here, in this house, for 20 years. The area used to be nice, but after the earthquakes [it changed]. It [other people’s houses] all got sold or rented [out] … so the neighbourhood’s changed [because friends and neighbours moved away]. Before [people moved away] I used to be quite chummy [friendly] with the neighbours (Participant 3).*

The second sub-theme of reflections identified how participants had reflected on a major change in their life course. As an example, one participant reflected on the loss of future social connection and planned activities after his wife died. *When my wife died, I didn’t seem to function. … It’s the end of the world. Why carry on? [We] were going to do so much [together] and it [didn’t happen] (Participant 9).* Another participant reflected how a personal health scare combined with observations about other people’s circumstances had made him aware of how isolated he would be if he could no longer continue working. *This business with the health thing … Lying on my hospital bed thinking about my life. … My life is my work. Outside of [work], I have absolutely nothing. No friends. I don’t drink. I don’t really do anything. Work is it. … If that goes, and at the same time I’m stuck somewhere [in a neighbourhood that does not have good transport and lots of neighbours and amenities] that’s it. … I have [become aware of] a lot of people who have been or still are in that situation [socially isolated], and I’m realizing I’m heading that way, and I don’t like the look of it (Participant 5).*

The final subtheme captured the resignation, coping or resilience that participants showed in relation to the aftermath of a major change in life course. Participants talked about how they had experienced a change and were no longer able to do something they loved, but did not appear to have made a plan to continue doing this by adapting their involvement in what had provided meaningful connection to them. *I used to show dogs. I started when I was in my early twenties. I had to stop showing them [because of my long-term condition]. I’ve just retired myself from judging … I don’t do it anymore [because of worsening symptoms of long-term condition] (Participant 2).* In contrast, other participants had a plan for connecting with others but appeared to lack the confidence to implement it. *I went to [an exercise class] a few times. It was way over there [other side of town] but then I broke my ribs and couldn’t exercise. I’d like to [go back] if it were closer. I did feel better after it. It gave me a bit of a social life. I was meeting people … I do have a number to ring, but I’m not sure if I can just book myself in (Participant 12).* Finally, some participants talked about the initial impact of being diagnosed with a long-term condition, but were also able to identify what they had done about this devastating situation. For example one participant had made connection with someone they knew and who they realized would be able to walk alongside them, and thus opened up a pathway for connection with other like-minded people. *I was wallowing quite badly [feeling depressed] for a while [after being diagnosed with a long-term condition]. … Then I organized to meet up with [a friend] at an exercise class. … [When I went] I thought I don’t belong here, they’re just a bunch of oldies [old people]. But I decided that I was just going to keep going [to the classes], and I’ve come to love those oldies (Participant 4).*

## 4. Discussion

The aim of this study was to explore with people with long-term conditions how they maintain and develop social connection, in order to be able to inform how healthcare professionals may assist people with long-term conditions to improve health and well-being. Three themes were identified. These were: “Meaningful connection”, “Wherewithal for social connection” and “Impact of a major change in life course”. 

The theme “Meaningful connection” captures three of the attributes of connectedness, those of intimacy, sense of belonging and reciprocity, outlined by Phillips-Salmi, Haase and Carter Kooken [14] in their concept analysis of connectedness. Concepts from the data were that participants sought meaningful connection for a variety of reasons, and that family, friends and neighbours provided a source of this for participants. Some of our participants also had meaningful relationships with people further afield through activities that they shared, for example at clubs, community activities, and including in voluntary or paid work. Meaningful relationships with others provides a feeling of belonging, with an associated improvement in health and well-being [30]. The notion of sharing of support with others in a reciprocal manner to the benefit of individual health and well-being has been explored in other research [15]. Indeed research suggests that giving support to others results in higher overall personal well-being, in contrast to the receiving of support from others [31]. This is because the giving of support and participation in society can facilitate development of a valued identity for the individual [21,31]. Therefore, for people with impairments arising from long-term conditions, a valued identity and valued life role would enhance individual well-being and alter perceptions of others of them being “unable”, or their own perceptions of themselves as being “disabled” by society.

The theme “Wherewithal for social connection” identified how intrinsic and extrinsic factors facilitated or impacted on the maintenance and development of social connection. Other research has identified the many factors that impact on the ability of individuals living with long-term conditions to socially connect with others [15,16,17,18,20,21,22,32] and our study has built on this by identifying how the factors contribute positively or negatively. Being able to maintain and develop social networks for meaningful connection and relationships contributes to development of social cohesion and subsequent building of social capital [30]. This potentially results in individuals and groups having access to knowledge, resources, support, and thus enabling them to live well through having power and control over their own lives within society [22,33]. Thus it could be argued that meaningful social connections and social capital are entwined and inextricably linked with the feeling of being valued, and therefore with health and well-being [34].

The theme “Impact of a major change in life course” encapsulated reasons why participants had experienced a major change and how this had influenced their ability to develop and maintain social connections. Cornwell [35] suggests that large social networks may provide individuals with the knowledge, resources and support (social capital) required for coping in times of crises or change. Equally, change resulting in loss of social connection and thus downsizing of networks may impact on access to the social capital “resources” available to the individual [15,36]. It is often at times of major change that individuals with long-term conditions come into contact with healthcare providers. This contact offers the provider an opportunity to build a caring, empathetic and respectful relationship with the individual and/or their family [14,37] that could extend beyond the reason for consultation. A scoping review by Mossabir, et al. [38] identified that social prescribing, where a healthcare provider suggests avenues such as community-based sources of support that may extend the individual’s social connectedness with others, would build social cohesion, networks and capital, and thus contribute to the individual’s overall feeling of psychosocial well-being [38]. Participants of the included studies in Mossabir et al.’s scoping review perceived they had better health status, and reported an increase in the frequency of social contacts and the number of leisure activities they attended, and reported an increase in volunteering and knowledge of local services. However, social prescribing to improve social connection for patients was reportedly labor intensive for the healthcare providers themselves [38]. Mossabir and colleagues [38] did point out that engagement in community-based sources of support was enabled by a “facilitator” on referral by a healthcare provider. The facilitator kept up to date with activities and services in the local community, and screened, assessed, referred and often accompanied participants to activities. This suggests that a meaningful relationship or connection with a trustworthy, empathetic and accessible “facilitator” is the key by which individuals with long-term conditions can be supported to begin attendance and thus link with community-based sources of support [38]. 

This study has both limitations and strengths. Firstly, a large number of participants had a primary diagnosis of chronic obstructive pulmonary disease, and thus, the findings may not be fully transferrable to people with other long-term conditions. However, our participants reported living with one to four long-term conditions each, with most participants reporting more than two conditions. Secondly, some participants were recruited through exercise groups for people with long-term conditions, and thus may be representative of a more proactive population who may be more socially connected. Therefore the findings again may not be representative of all people with long-term conditions. Thirdly, this study did not explore whether mental illness or socio-economic status (which we did not specifically ask about) were contributing factors to participants’ feelings and experiences. The strengths of the study were that the trustworthiness of the study’s findings was upheld through multiple discussions by the research team, where ideas for themes were brought forward, discussed and reiterated until a group consensus was reached [39,40]. In addition, the cross-checking of data and the continuation of data analysis past saturation further improved the trustworthiness of the findings [41]. Despite the limitations of qualitative research, we believe our study provides contextual knowledge for further research about the role that connections with friends, family and community members can have in helping people with long-term conditions feel that they belong and have a role to play in society.

## 5. Conclusions

The findings from this study suggest that social connection is valued by people with long-term conditions. Social connection facilitates meaningful ways to give and receive support, and thus provides access to knowledge and resources for better health and well-being. People with long-term conditions experience challenges to developing and maintaining social connectedness in their everyday life, especially after a major change in life course. We suggest that healthcare providers are well placed to encourage, explore and facilitate ways for people with long-term conditions to socially connect with others in their neighbourhood and community, and that this in particular be attended to after a major life change. Moreover, it appears that such “social prescribing” may be best achieved through referral to a trustworthy, empathetic and accessible “facilitator”. Indeed, referral by healthcare professionals to such facilitators may be a key way by which individuals with long-term conditions can be supported to link with community-based sources of support.

## Figures and Tables

**Table 1:** Semi-structured interview questions.

Demographics: age, gender, long-term condition diagnoses, employment status, number of people in the home	
Q1	Who are the people in your life that are important to you? How do you keep in contact with them (for example, by phone or computer)? If house bound – which people come to your home? In what ways do you feel connected to family, friends, neighbours, and your community? Why and how?
Q2	What does a normal week look like for you? What do you like to do for yourself (for example, your hobbies/interests/activities)? Can you tell me more about (activities attended)?
Q3	How do you get out and about in your neighbourhood? For example, getting to the doctors, shops, and supermarket.
Q4	What does the term community or neighbourhood mean to you? Prompt questions - What is it like living in this community? What do you enjoy/dislike about living in this community? How long have you lived here in this home/neighbourhood?
Q5	What does the term “social connectedness” mean to you? Prompt question - compared to social interaction.

**Table 2:** Demographic information for study participants.

Participant Number	Age	Gender	Diagnoses
P1	50	F	Multiple sclerosis
P2	71	F	COPD
P3	69	F	COPD
P4	59	F	COPD, osteoarthritis
P5	66	M	Heart disease, COPD
P6	54	F	COPD, rheumatoid arthritis
P7	56	F	Bipolar disorder, type 2 diabetes mellitus, Parkinson’s, COPD
P8	59	F	Type 2 diabetes mellitus, COPD, gastro-oesophageal reflux
P9	64	M	COPD
P10	74	F	COPD
P11	79	F	COPD
P12	61	M	COPD, depression
P13	72	F	COPD
P14	69	M	COPD
P15	71	F	Type 2 diabetes mellitus, COPD, osteoporosis
P16	78	M	COPD, prostate cancer
P17	52	F	COPD, osteoporosis, fatty liver disease

Abbreviations: COPD—chronic obstructive pulmonary disease.

**Table 3:** Summary of themes, subthemes and categories.

Themes	Subthemes	Categories
Meaningful connection	For a reason	Common interest Helping others I need assistance It is good for me
	With family, friends, neighbours & others	
Wherewithal for social connection	Intrinsic person factors	Mind-set Symptoms of condition Ability to use/dislike of using technology to connect with others
	Extrinsic factors	Finance Weather Proximity to amenities
Impact of a major change in life course	Reasons for major change	Retirement Major illness or diagnosis or physical/mental effect of long-term condition Natural disaster Death of a spouse
	Reflections	On own loss On others’ disconnected lives
	Resignation, resilience and coping	No plan to continue with a meaningful connection by adapting one’s involvement Have a plan but lack confidence to implement it Making a plan and putting it into action
